# New windows into retroviral RNA structures

**DOI:** 10.1186/s12977-018-0393-6

**Published:** 2018-01-25

**Authors:** Dhivya Jayaraman, Julia Claire Kenyon

**Affiliations:** 1 Department of Medicine, National University of Singapore, 14 Medical Drive, MD 6, Level 15, Singapore, 117599 Singapore; 2 Department of Medicine, University of Cambridge, Level 5 Addenbrookes Hospital Hills Rd, Cambridge, CB2 0QQ UK; 3 Department of Microbiology and Immunology, National University of Singapore, 5 Science Drive 2 Blk MD4, Level 3, Singapore, 117545 Singapore; 4 Homerton College, University of Cambridge, Hills Rd, Cambridge, CB2 8PH UK

**Keywords:** Retrovirus, RNA structure, Secondary structure, Disruptive technology, SHAPE, NMR, HIV

## Abstract

**Background:**

The multiple roles of both viral and cellular RNAs have become increasingly apparent in recent years, and techniques to model them have become significantly more powerful, enabling faster and more accurate visualization of RNA structures.

**Main body:**

Techniques such as SHAPE (selective 2’OH acylation analysed by primer extension) have revolutionized the field, and have been used to examine RNAs belonging to many and diverse retroviruses. Secondary structure probing reagents such as these have been aided by the development of faster methods of analysis either via capillary or next-generation sequencing, allowing the analysis of entire genomes, and of retroviral RNA structures within virions. Techniques to model the three-dimensional structures of these large RNAs have also recently developed.

**Conclusions:**

The flexibility of retroviral RNAs, both structural and functional, is clear from the results of these new experimental techniques. Retroviral RNA structures and structural changes control many stages of the lifecycle, and both the RNA structures themselves and their interactions with ligands are potential new drug targets. In addition, our growing understanding of retroviral RNA structures is aiding our knowledge of cellular RNA form and function.

## Background

A recent wave of RNA-structure related techniques has brought new and different ways in which we can view retroviral RNAs in two and three dimensions, both inside and outside cells. Such technical power has shown us that retroviral RNAs are physically and functionally flexible and multidimensional, and insights that emerged from these studies initially are now enabling wider understanding of cellular RNA form and function.

### Techniques to map two-dimensional structures in RNA

**Fig. 1:**
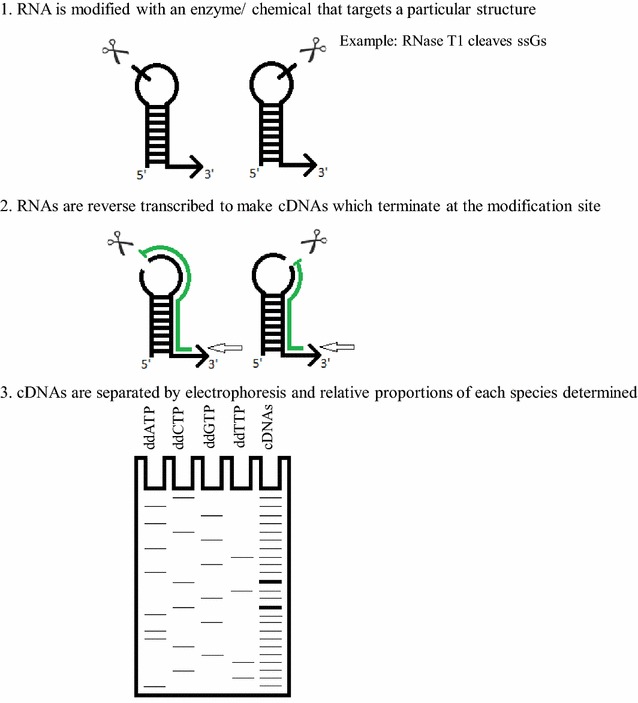
The basic principle of RNA structure probing

Less than a decade ago most RNA structure probing was performed using enzymes and chemicals with limited reactivity, specific for particular bases and structural motifs. These reagents were used to cleave or modify RNAs that were either radioactively end-labelled, or subsequently reverse transcribed into radiolabeled cDNAs, to map the modification sites (Fig. 1) [1, 2]. For example, RNase T was frequently used to target single-stranded guanosines, and low concentrations of RNAse A were used to cleave 3’ of single-stranded pyrimidines. Double-stranded RNA could be probed with CV1 enzyme, which tends to cleave only towards the middle of longer, more stable helices; and so more often, double-stranded regions were detected by the absence of reactivity of a combination of several of the single-strand targeting reagents [3]. These, however, all displayed substantial nucleotide or specific structural bias, thus necessitating the use of a number of different reagents to examine one RNA structure. The resolution of such experiments was similarly time-consuming, using denaturing sequencing gels onto which radioactive cDNAs or end-labeled RNAs were loaded. This was technically challenging and laborious, rendering the study of an RNA as long as the retroviral genome prohibitively slow. However, major advances in probing reagents and sample resolution techniques reformed the field, making RNA secondary structural analysis on the viral genome scale, or analysis of multiple mutants or conditions possible.

### Finer resolution mapping of RNA structures: SHAPE reagents

SHAPE (selective 2’OH acylation analyzed by primer extension) reagents selectively acylate the ribose 2’hydroxyl, where the backbone is flexible [4]. This corresponds mainly with single-stranded regions. One major advantage of using SHAPE reagents is that they react with each nucleotide irrespective of base; they are far more sensitive to the structural context the base is in [5]. These reagents quickly helped to refine various retroviral RNA structures that had previously been characterized by enzymatic probing [6, 7]. Reverse transcription of SHAPE reagent-modified RNAs leads to a truncated cDNA as the RT enzyme cannot polymerise past the acylation site under standard conditions [4]. Users can thus probe RNA with just one reagent, instead of a combination of several different reagents, which quickly builds a more accurate picture of RNA secondary structure. Single-stranded nucleotides are reactive to SHAPE reagents unless the backbone is constrained through stacking or noncanonical interactions. As these sorts of interaction tend to be limited to individual or small stretches of contiguous nucleotides, longer stretches of the RNA that lack acylation sites can signal base-paring that was not predicted computationally using standard minimal free energy modeling algorithms [8]. Such algorithms don’t take into account higher-order structures such as pseudoknots and intermolecular interactions. These potential interactions can be further examined by mutation to distinguish whether the lack of SHAPE reactivity is indeed due to higher-order interactions, or whether the original model on which they were based needs to be modified. The signal coverage achieved by SHAPE reagents also decreases the number of experiments necessary in order to inform structural modeling effectively. Thus, users are able to screen for the structural effects of mutations on the native RNA structure quickly and more accurately than before. This is particularly useful when working with multiple lengths of RNA [8, 9] or with large RNAs like the Rev Response Element (RRE). Using SHAPE reagents, Legiewicz and co-workers were able to investigate the RRE structures that were resistant and susceptible to Mov10, an inhibitor of RNA export [10]. Analyses of the dynamics of the Rev–RRE interaction also became possible because the RNA structure could be examined at multiple points during the refolding process [11]. When probing large cellular or virion RNAs that are not overly abundant the coverage given by SHAPE reagents is also very important as it requires fewer experiments in order to ascertain the structural environment of each nucleotide, and hence requires less RNA to be made and purified. This enabled the structural mapping of the entire HIV-1 genome isolated from virions [12].

### Faster mapping of RNA structures: capillary electrophoresis

**Fig. 2:**
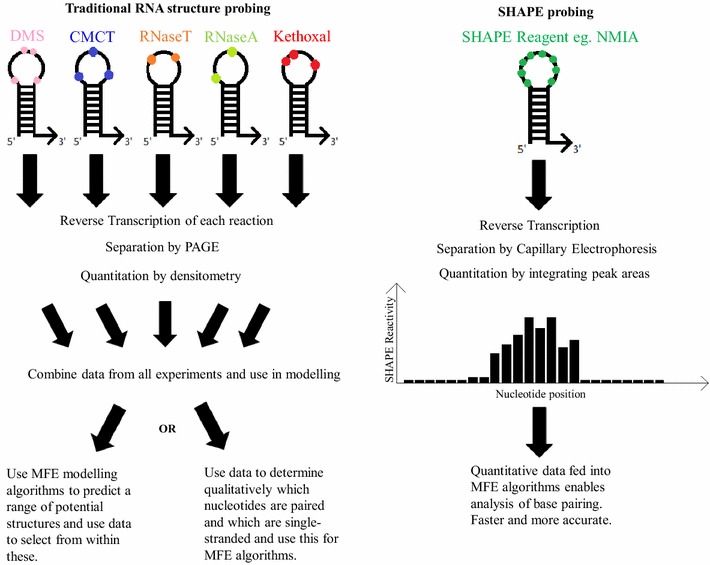
Traditional probing versus high-throughput SHAPE probing. SHAPE reagents streamline and improve the RNA structure probing process. Structure probing reagents are used at limiting dilution, where there is up to one modification per RNA molecule. All potential sites of modification are shown for each reagent. The left hand panel illustrates structure probing with traditional, more specific, reagents, and analysis by gel electrophoresis. The right hand panel shows structure probing with SHAPE reagents and analysis by capillary electrophoresis. It should be noted that it is possible to use either analysis technique for any RNA structure probing reagent

Resolving techniques for cDNAs made during primer extension assays have also changed the field. Users have moved from manually pouring sequencing gels and running radioactively labeled samples, followed by analysis by densitometry or by eye, to separation by capillary using fluorophore-labelled cDNAs and more integrated analysis [13]. This allows higher-throughput, less margin for human error and a more automated alignment and measurement of amount of cDNA at each position (Fig. 2). This analysis method has revolutionized the study of RNA secondary structure not only by SHAPE reagents but also other probing reagents such as RNases, DMS and hydroxyl radicals [14]. The real strength of this method is the ability to run multiple samples (commonly 96) at once, meaning that if the RNA to be probed is abundant, multiple replicates can be performed in parallel, sampling different experimental conditions.

## What have these techniques shown us for retroviruses?

The functionality of both SHAPE and capillary electrophoresis have enabled analyses of many distinct retroviral RNA structures, from lentiviruses like HIV-1, HIV-2 [8], SIV [15] and FIV [7], to MPMV [16], MMTV [17], MoMLV [18] and foamy virus [19]. In some cases, SHAPE reagents have helped to identify specific long range interactions and dimerization initiation sites [7, 17] that are hallmarks of retroviral RNA folding and behavior [20].

Perhaps the most important and surprising property of retroviral RNA structures that has been uncovered by the new wave of RNA secondary structure probing techniques is the degree of their flexibility. Where RNA sequences are duplicated within the genome or between genomic and spliced RNAs, it is becoming apparent that the different RNA structures formed by the same RNA sequence give it completely different functional properties. For example, polyadenylation and splice donor site choice between 5’ and 3’ ends of the retroviral genome have been observed to be due to the formation of different RNA structures for foamy viruses [21]. RNA transcribed from the 5’LTR folds to expose the major splice donor (mSD) site to the U1snRNP, which suppresses polyadenylation at this site, whereas at the 3’UTR the RNA fold sequesters the mSD from binding to the U1snRNP, leading to RNA cleavage and polyadenylation. The authors were able to gain a more in-depth understanding of the biological roles of each part of the RNA structure by examining the RNA structures of mutants in addition to wild-type viral sequences; such comparisons would have been prohibitively slow using previous technology. SHAPE data have also been used to show a link between the structural context of the 5’ mSD site in HIV-1 and the efficiency of genome dimerization [22]. Within one specific retroviral RNA sequence, though, it appears that there can be considerable structural variation. The many different models for HIV-1 dimerisation, some observed even within the same group, may be a reflection of the ability of this RNA to remodel multiple times [23, 24]. Indeed, it was recently shown that it is nearly impossible to introduce mutations into the RNA leader that have no effect on its structures, even in the single stranded regions [25]. Many groups have examined RNA structural changes using mutational analyses; stabilizing or preventing predicted structures from forming. However, as the number of plausible structural models for each individual RNA sequence increases it is becoming likely that many retroviral RNAs exist in a fine balance between several different structures, and that introduced mutations may have unpredictable off-target effects on some of these, making it harder to identify all structures within the population. We recently developed a technique that avoids this concern by examining RNA structures within a native gel matrix [26]. In-gel SHAPE separates conformers of differing electrophoretic migration by native acrylamide gel electrophoresis. The gel piece containing each conformer is then physically isolated and soaked in SHAPE reagent, before recovering the RNA. Using this technique a structural switch between monomeric and dimeric HIV-1 packaging signal RNA was instantly apparent, as the  dimerisation initiation site (DIS) was unreactive in monomeric RNA as well as in dimeric RNA. In-gel SHAPE has subsequently been used to examine two conformers of the HIV-1 RRE that had been structurally inseparable using atomic force microscopy [27]. The authors of the SHAPE study then used the in-gel SHAPE results to design mutations that disrupted the Rev–RRE interaction [28]. Even when probing in solution SHAPE reagents can indicate the presence of multiple structures. Although the structure of Moloney murine sarcoma virus RNA derived using SHAPE probing was very similar to previous models, in one region a number of partially reactive nucleotides signified the presence of multiple structures, which the authors were then able to model [29]. The relatively even reactivity of SHAPE reagents with all four ribonucleotides can also help to distinguish and model alternative structures more accurately when probed in solution, by taking into account the proportion of RNA in each conformation and separating the overall SHAPE signal coming from all structures in the population into theoretical individual signals coming from the individual structures [11], on the basis that these must add up to give the overall SHAPE reactivity value. The more global mutagenesis technique mutate-and-map [30] may also prove useful for understanding the balances of structures present, as it has been applied to the study of riboswitches.

The flexibility of retroviral RNA does not necessarily limit it to a finite number of distinct structures, but also enables it to remodel during interactions with proteins or other ligands. The fast-acting SHAPE reagent benzoyl cyanide has been used to study the folding pathway of the RRE as it interacts with increasing amounts of the Rev protein [31]. Conversely, by identification of RNA sequences or regions with low SHAPE acylation sensitivity and low Shannon entropy, SHAPE reagents have also been used to pinpoint retroviral RNAs that adopt a single, stable fold. This has been observed for various regions of the HIV-1 genome [32] and for the RNA leader of HIV-2 in the loose dimer state [8].

### Functional mapping of RNA structures

The study of RNA structures is incomplete without an understanding of their functions. Retroviral RNAs interact with host and viral RNAs and proteins, and several techniques to map and model interaction sites have been recently developed and applied to retroviruses. CLIP data will be discussed elsewhere in this edition and hence will not be discussed here. MIME (mutational interference mapping experiment) is an in vitro approach, using protein ligand to capture mutated RNAs and Illumina sequencing to determine which mutated sequences retain the ability to bind to the ligand, and which mutations prevent binding [33]. This approach highlighted the structural importance of SL1 to the Gag-binding process in HIV-1, as well as the defining the region containing the optimum RNA structures for Gag interaction as being nucleotides (nts) 227–337.

RNAs, and particularly those of retroviruses, often have the ability to switch structure in order to interact with different cognate ligands. One disadvantage of using chemical or enzymatic probing agents to ‘footprint’ RNA-ligand interactions is that the disappearance of signal could indicate the presence of ligand blocking the access of the reagent, or it could indicate an RNA secondary or tertiary structural change, which may be distant from the ligand binding site. XL-SHAPE combines identification of RNA structural changes using N-methylisatoic anhydride (NMIA), with protein binding site identification using cross-linking [34]. The technique has been used to model the Gag binding sites and ensuing RNA structural changes in the HIV leader RNA during the packaging process. Of course, neither MIME nor XL-SHAPE techniques provide direct information on the RNA–protein interactions necessary to form a mature, infectious virion, and data from in vivo techniques such as CLIP may not capture each of the RNA–protein interactions that occur during virion formation and maturation as some of these may be transient. It may be that the greatest achievements in understanding retroviral RNA structure–function relationships will come from a combination of in vitro and in vivo techniques.

SHAPE has also been combined with evolutionary analyses to identify conserved RNA structures of unknown function: low acylation sensitivity of a region indicates that it is likely to be structured. Zanini et al. [35] used published SHAPE data and structural models to show that synonymous mutations that would destabilise the low SHAPE-reactivity RNA helices flanking the variable loops of HIV-1 gp120 are selected against, indicating the presence of an important RNA structural signal, and further structures exist elsewhere in the HIV-1 genome [36]. Evolutionary and SHAPE analyses have also been combined to pinpoint conserved RNA structures with greater accuracy than using computational methods alone. A comparison of HIV, SIVmac and SIVcpz genomes identified previously known functional elements, alongside many that were previously unidentified [37].

RNA structures identified can be examined functionally by mutation and viral replication assay. In many cases, such mutations have provided confirmation of their importance to the viral lifecycle. For example, a stable RNA stem-loop observed by SHAPE in Rex-dependent but not in Rex-independent human T-lymphotropic virus RNAs was subsequently found to promote nuclear retention and mRNA degradation, aiding the virus to temporally control its gene expression [38]. However, sometimes RNA structures that have been modeled using both SHAPE data and evolutionary analyses are not found to have an important role in viral replication, at least in cell culture [39].

### In-cell and in-virion mapping of secondary structures

SHAPE reagents, alongside some of their longer-established counterparts, diffuse across membranes and can thus be used to probe RNA structures inside living cells. Such data are then examined by various next-generation sequencing methods, and have begun to show fascinating insights into the cellular regulation of RNAs, and cellular regulation by RNAs. The structures of retroviral RNAs inside living cells have not yet been established, perhaps because of their enormous structural flexibility and low abundance, but technology to enable this has developed so rapidly that it is likely to be a relatively short time before we begin to see these results [40–42].

Several studies exist however that examine the structures of retroviral RNAs inside virions (*in virio*). An early study using RNAs prepared in different ways found little structural difference between in vitro transcribed, *ex virio* and *in virio* structures of the HIV-1 leader [13]. Seif and colleagues examined the structures of wild-type and protease defective virions, and detected differences in SHAPE reactivity in the upper PBS stem and PBS internal stem-loop, enabling them to propose that Gag promotes partial annealing of tRNA to the HIV-1 PBS, with Ncp7 then catalyzing a more extensive annealing as the virion matures [43].

*In virio* and *ex virio* data are also beginning to be used to inform different aspects of retrovirology. Such data have been used to provide evidence for new models of HIV-1 dimerisation [44] as well as to understand the driving force for positive selection in HIV. Relative to other determinants, such as evasion of antibody or T cell responses, the maintenance of RNA structure was found to be the dominant factor [45].

RNA modifications such as 6-methyl adenosine (m6A) have recently been linked to control of RNA function. In HIV-1, m6A deposition was shown to be vital for viral RNA expression and function [46], although the role of RNA structure in modified RNAs is yet to be determined.

### Structural routes to new therapies

The ability to examine RNA structure and structural changes so quickly has had knock-on effects on the development of therapeutics. Recently, SHAPE has been used to identify structural effects of small molecules that bind specifically to HIV-1 TAR RNA [47] and to SL3 (J Kenyon, unpublished data). Monitoring the structural perturbations caused by RNA binding drugs helps to understand their mechanism of action and also helps to ensure their specificity. When working with therapeutic RNAs rather than drugs, understanding the structure of the target HIV-1 RNA has proved to be extremely valuable. The efficacy of inhibitor shRNAs was found to correlate with high SHAPE reactivity in both the seed region and a downstream flanking sequence, and the authors were able to apply this knowledge to their shRNA design, choosing targets in which these sequences were structurally accessible, and resulting in improved success of this strategy [48]. The cellular process of trans-splicing has also recently been harnessed to couple an inducible lethality gene onto HIV-1 transcripts [49]. *Trans*-splicing, in which the splice donor and acceptor sites are initially on separate RNAs which are subsequently joined occurs naturally within cells, albeit to a much lesser extent than the constitutive *cis*-splicing process. Once again, the authors used published SHAPE reactivity information to identify RNA sequences whose structures would be most likely to promote *trans*-splicing. This approach resulted in a high degree of success, with proof of principle that multiple sites in the retroviral genome are targetable using this approach.

### Three dimensional techniques

Structural analysis of retroviral RNA is a compelling necessity while designing synthetic drugs against specific molecular targets within it. And with steady RNA structural changes adopted to suit different functions throughout the viral life cycle it is important to understand the spatial orientation of the various RNA structural domains, and their structural flexibility. Single molecule FRET (smFRET) can be performed on long RNAs but requires only small quantities of sample to predict native sequences in their physiological conditions [50]. It relies first on SHAPE data to predict 2D stuctures, which are then interpreted in 3D using smFRET-derived distance constraints. It works on the principle of energy transfer through dipole–dipole interactions between the donor and acceptor fluorophores conjugated to the RNA at specific locations. It can help to predict the intramolecular interactions with high resolution and helps to understand the complicated design of 3D conformational changes. The technique, coupled with molecular modeling, has been used to visualise the PBS and packaging signal domains of HIV-1 [51]. This showed a conserved kink turn motif, revealing a potential protein binding site that may facilitate Gag binding during RNA genome packaging and subsequent tertiary structural changes. Small Angle X-ray Scattering (SAXS) also models large biomolecular structures. As it does this based on their electron density it can be done without coupling fluorophores to the RNA, although it, too, relies on prior secondary structural prediction using SHAPE or similar techniques [52].

A SAXS study of the HIV-1 5’ UTR revealed that the PBS fold displays molecular mimicry of tRNA [53]. SAXS has also been employed to elucidate the tertiary structure of the RRE and to understand the dynamics of Rev binding, showing a two-step model involving initial sequestration of some Rev binding sites until the RRE has bound Rev at a group of exposed sites, causing it to switch structure and exposing a further binding site [31]. SAXS has also been used to throw more light on the RRE’s nuclear export role of transporting unspliced and partially spliced RNAs [54]. Hydroxyl radical cleavage experiments have also been used to investigate the three-dimensional architecture of retroviral RNAs, showing that secondary structural elements pack together rather than acting independently [55].

Of the methods that provide the greatest structural detail, enhancements in experimental NMR techniques have recently expanded the size of retroviral RNA structures able to be visualized. This is partly due to the implementation of ^1^H–^13^C correlated HMQC NMR, whereby short stretches of ^13^C-edited RNA are ligated to non-edited RNA, allowing the observation of signals that can be assigned to the ^13^C edited RNA segment. Using this technique along with Adenosine interaction detection, the Summers lab proposed the monomer–dimer structural switch that was subsequently seen using in-gel SHAPE [56]. Although not performed on as large a section of RNA, ^2^H-edited NMR was also used by the same laboratory to assign NMR signals to specific residues and identify a three-way junction that the RNA is able to adopt, flattening SL2 [57]. ^2^H editing was also recently used to show that the dimerization contacts between strands are much more extensive than previously thought, extending beyond SL1 and to the U5: AUG region [58]. This raises intriguing possibilities about the structural maturation of the RNA after dimerization is initiated. X-ray crystallography examines static RNA structures, hence this technique has been used to examine the structural basis of nucleic acid- protein complexes, rather than large and flexible RNA molecules [59]. Although cryo-electron microscopy has so far been used in the retrovirus field mainly for the study of capsids [60] the incredibly fast moving nature of this field, as well as the success of current studies on large RNA–protein assemblies such as the spliceosome [61] suggests that this technique may soon provide RNA structural models for retroviruses.

### Working with structure probing reagents: which to choose?

SHAPE reagents will all react with the ribose 2’OH according to backbone flexibility, and are similarly insensitive to the base composition. The reaction kinetics vary between reagents, with the time the reagent is active for, before it is hydrolysed from milliseconds (benzoyl cyanide [62]) to hours (2-methyl-3-furoic acid imidazolide (FAI) [42]), and this is where the user must consider the experiment: for in vitro experiments in solution following dynamic changes in the RNA, BzCn will be the most informative reagent, when used to perform a series of timed snapshots of the RNA conformation. However, when probing inside cells the reagents with longer half-life have been shown to enable better probing, particularly with less abundant transcripts [63]. 1-methyl 7-nitroisatoic anhydride (1M7) and NMIA have intermediate half-lives and are often used to probe in vitro transcribed structures. Finally, if users wish to map structure on a finer level, including noncanonical interactions, the acylation profiles of NMIA and 1-methyl-6-nitroisatoic anhydride (1M6) can be compared. This can be useful not only for de novo analyses of retroviral RNA structure, but also to verify that experimental conditions used have not altered RNA structure, as was performed when examining the HIV-1 frameshift RNA structure [64]. Other biochemical or enzymatic probes can be used to further examine the RNA structure, with DMS able to identify some noncanonical interactions, and hydroxyl radical probing used to examine solvent accessibility of the backbone regardless of secondary structure [65]. A recent observation that there is substantial nucleotide bias in single- stranded regions of retroviruses, such as the presence of more than 50% As in single-stranded regions of HIV-1, means that if probing with a second type of reagent, an appropriate one can be chosen- in the case of HIV-1, DMS would seem appropriate [66]. Whichever reagent(s) are chosen, they should first be titrated to ensure that the concentration the user is working with results in approximately one modification per RNA molecule, and not more.

### Overcoming the limitations of SHAPE reagents

Although SHAPE reagents provide detailed information on the flexibility of the RNA backbone, interpretation of which nucleotide base pairs with which is generally inferred computationally, using minimal free energy modeling and programs such as RNAstructure [67]. To gain further insight into long-range interactions or alternative foldings of the RNA, techniques designed to perturb possible interactions can be employed. Antisense interfered SHAPE uses modified oligonucleotides such as LNAs that have higher affinity than their natural counterparts for the native RNA. The antisense oligos are designed to outcompete a proposed native intermolecular interaction, rendering one side single-stranded. This is seen as an increase in backbone flexibility by enhanced SHAPE reagent binding, and was applied to HIV-2 [8] and alternative foldings in HIV-1 [22], to identify different types of long-range interactions in the murine musD retrotransposon transport element, [68], as well as to identify a long-range interaction formed from noncanonical pairings in the Rev Response Element [31]. All of these accomplishments would have required prohibitively laborious experiments if done using conventional probing reagents. The natural sequence variation found in retroviral RNAs can also guide structural modeling and the design of mutants that will not perturb the overall RNA structure [69].

### Resolving and analyzing cDNAs by capillary electrophoresis: designing an experiment around available equipment

The limiting factor in capillary electrophoresis is likely to be the availability of an appropriate capillary sequencing machine and experiments may have to be tailored around the make and model to be used. Several programs are available for analysis; some of these necessitate the use of particular fluorophores. The first of the analysis programmes to be established, SHAPEfinder software is Mac-specific and is rigid in its interpretation of each wavelength, necessitating the use of sets such as 6FAM, VIC, NED and PET on an Applied Biosystems instrument, to run experimental and control samples and two sequencing ladders respectively [70]. However, by using four different fluorophore channels per capillary experiment, fewer sequencing wells can be used. In contrast, alternative software packages such as QuSHAPE use only two fluorophore channels per capillary experiment, separating the SHAPE-modified cDNA and the unmodified control cDNA into two separate wells, each including one fluorophore channel as a ladder [71]. This enables the user to work with fewer fluorophore-labelled oligonucleotide primers at the expense of performing more capillary separations. This is particularly beneficial when examining a large RNA using multiple different primers, as users normally work with around one primer per 300 nt. A third recently developed analysis programme, RiboCAT, uses MS Excel and is thus more adaptable to different hardware [72]. Alternative programs are aslo available [73, 74]. When it comes to preparing the ladders for alignment of cDNA fragments with the nucleotide sequence they represent, several different strategies can be used. If RNA is abundant, ladders can be manufactured by reverse transcription, incorporating a small proportion of defined ddNTPs [13]. When RNA concentration is limiting however, a sequencing ladder can be performed from a DNA template using cycle-sequencing [7]. If the cycle sequencing ladder uses 7-deazadGTP to eliminate G-compressions which would otherwise distort the ladder, 7deazadGTP can also be incorporated during cDNA manufacture from SHAPE reagent probed RNAs [7]. Alternatively, it is possible to work with standard size markers for capillary electrophoresis [72]. Although the great beauty of this technology is in its wide applicability due to the relative abundance of standard capillary sequencers, it is possible to adapt the sequencing equipment so that it is ultra-sensitive and can be used to examine femtomolar quantities of RNA [75]. Using this equipment, the same group examined differences in MoMLV gRNA structure inside immature and mature virions, showing that the immature virion contains RNA in the structure of a dimerization intermediate.

### Drawbacks of capillary electrophoresis experiments

Capillary electrophoresis is commonly used to study molecules of over 50 nt in length. This is particularly if using primer extension, as is used to map acylation sites of SHAPE reagents. However, techniques exist to map SHAPE reagent binding to smaller RNAs also, such as SHAMS, which uses mass spectrometry to pinpoint acylation sites on small RNAs [76] and selective 2’OH acylation analysed by protection from exoribonclease [77].

## Conclusions

The understanding of both viral and cellular RNA structures is becoming more vital for our understanding of retrovirology in general, as the viral roles of RNA structure become more apparent. These disruptive technologies give retrovirologists the power to investigate and visualize such structures with relative ease. From distinct types of retrovirus it is clear that RNA structures are as vital to the lifecycle as protein structures, with viral protein function often dependent on viral RNA structure. The TAR-Tat interaction of HIV-1 has been understood for many years, but the disruptive technologies discussed in this article have established the importance of further interactions across retroviral genomes and genera.

Moreover, as the multiple roles of RNA in both viral and cellular processes become apparent, our growing knowledge of retroviral RNA behaviour helps to understand their cellular counterparts. For example, an RNA stability element (RSE) in Rous sarcoma virus that maps to a region downstream of the Gag stop codon was examined by SHAPE and by mutational analyses, to determine the mechanism by which it prevents nonsense mediated decay (NMD) of the viral genomic RNA. The authors found that the RNA structures mediating this effect were separated at defined distances from one another within a 155 nt region of the RNA, and proposed that this overall RSE structure acts as an insulator from the NMD machinery. Their results added weight to the argument that the exon junction complex is not required in order to identify a premature stop codon [78], and were followed up by an analysis of cellular binding partners of the RSE, identifying polypyrimidine tract binding protein 1 as the factor shielding both retroviral and cellular RNAs from the NMD machinery [79]. Such interesting windows into cellular RNA function are likely to widen as we pursue structural studies of retroviral RNAs.
